# Immuno−oncological effects of aerobic exercise combined with anti−PD−L1 antibody blockade in a murine breast cancer model

**DOI:** 10.3389/fonc.2026.1671244

**Published:** 2026-01-27

**Authors:** Amirhossein Ahmadi Hekmatikar, Hamid Agha-Alinejad, Aliakbar Yousefi-Ahmadipour, Mahdieh Molanouri Shamsi, D. Maryama Awang Daud

**Affiliations:** 1 Department of Sport Science, Faculty of Humanities, Tarbiat Modares University, Tehran, Iran; 2 Immunology of Infectious Diseases Research Center, Research Institute of Basic Medical Sciences, Rafsanjan University of Medical Sciences, Rafsanjan, Iran; 3 Molecular Medicine Research Center, Research Institute of Basic Medical Sciences, Rafsanjan University of Medical Sciences, Rafsanjan, Iran; 4 Department of Laboratory Sciences, Faculty of Paramedicine, Rafsanjan University of Medical Sciences, Rafsanjan, Iran; 5 Cancer and Stem Cell Research Laboratory, Faculty of Paramedicine, Rafsanjan University of Medical Sciences, Rafsanjan, Iran; 6 Health Through Exercise and Active Living (HEAL) Research Unit, Faculty of Medicine and Health Sciences, Universiti Malaysia Sabah, Kota Kinabalu, Sabah, Malaysia; 7 Department of Biomedical Sciences, Faculty of Medicine and Health Sciences, University Malaysia Sabah, Kota Kinabalu, Sabah, Malaysia

**Keywords:** anti-PD-L1 antibody, breast cancer, CD4 and CD8, physical activity, tumor volume

## Abstract

Immunotherapy has emerged as a crucial approach in cancer treatment, particularly through targeting immune checkpoints such as programmed cell death protein 1 (PD−1) and its ligand programmed cell death 1 ligand 1 (PD−L1). Blocking antibodies against PD−1/PD−L1 have demonstrated the potential to activate tumor−specific immune cells, particularly CD4^+^ and CD8^+^ T cells. Recently, research in exercise oncology has underscored the role of physical activity in augmenting immune function in cancer settings. This study explored the combined impact of aerobic exercise and anti−PD−L1 antibody administration on immunological and physiological responses in a murine breast cancer model. Materials and Methods: Thirty female BALB/c mice were divided into five experimental groups: Patient control group (n=6), Exercise+induction+ control (n=6), Exercise+induction+exercise (n=6), Exercise+induction+anti-PD-L1 (n=6), and Exercise induction exercise + anti-PD−L1 (n=6). After a treadmill acclimation period, mice underwent two 6-week initial training protocols, followed by an additional 4-week protocol post-tumor induction. Following tumor induction, the PD−L1anti−PD−L1 antibody was administered. One-way analysis of variance (ANOVA) was applied to evaluate experimental variables. Results: Statistical analyses showed that, relative to the PCG, mice receiving the combined intervention of EIE and EIE + A displayed higher intratumoral CD4^+^ and CD8^+^ T cell densities and smaller final tumor volumes. Similar but less pronounced trends were observed in the EIE and EIE+A. Conclusion: Within the limits of the current design, the combination of aerobic exercise with PD−L1 immune checkpoint blockade was associated with increased tumor−infiltrating CD4^+^ and CD8^+^ T−cell density and reduced tumor burden. These findings suggest a potential interaction that warrants evaluation in future studies incorporating a PD−L1−monotherapy group to clarify independent and combined effects.

## 1 Introduction

Despite decades of laboratory, epidemiological, and clinical research, the incidence of BC continues to rise, attributed to inactive lifestyles, poor nutrition, stress, and genetic factors (1). BC remains the leading cause of disease burden among women, affecting one in every 20 individuals globally and one in every eight individuals in high-income countries (1). Numerous treatment methods for BC have been identified; however, the side effects of these treatments remain unavoidable (2). The complex nature of cancerous tumors has made cancer treatment challenging, and immunotherapy has emerged as a promising approach in addressing cancerous tumors (3, 4). Immunotherapy focuses on inhibiting two immune system regulators: PD-1 and PD−L1 (5). Within the tumor microenvironment, PD-1 and its ligand PD−L1 play critical roles in tumor progression and survival by evading immune surveillance. PD-1 is expressed on various immune cells, including monocytes, T cells, B cells, dendritic cells, and tumor-infiltrating lymphocytes (5). In the cancer immune cycle, the immune checkpoint PD-1 and its ligand PD−L1 collaborate to help tumors resist immune-induced apoptosis and promote tumor progression (5). PD−L1 is expressed on tumor cells and APCs, and its binding to T cell PD-1 disrupts T cell function, preventing their infiltration into the tumor (5, 6). Thus, tumors expressing PD-L1 can evade antitumor immune responses by engaging PD-1 on T cells (5, 6). CD4+ and CD8+ are tumor-specific immune cells crucial for cancer cell death and tumor destruction. Recent studies have reported that blocking PD−L1 with anti-PD−L1 antibodies can be a promising therapeutic strategy for treating cancerous tumors (7, 8). These positive outcomes are attributed to enhanced CD4+ and CD8+ function and their increased penetration into the tumor (7, 8). This treatment has shown significant results when combined with chemotherapy, although the side effects of chemotherapy have limited its consideration by researchers. Nonetheless, developing safe strategies alongside immunotherapy could offer promising advancements in cancer treatment.

Examining the history of exercise immunology reveals that PE can be a pivotal and safe means of enhancing the body’s immune system function (1). The enhancement of immune system function through PE has been particularly noted in the immunological changes of the adaptive immune system, specifically the improvement of T cell function (1, 2). In recent years, the relationship between PE and cancer has garnered significant attention from exercise oncology researchers, with a focus on the immunological changes induced by PE in cancer patients (3). A study highlighted the crucial immunological benefits of PE for BC patients, suggesting that integrating PE with immunotherapy could pave the way for safer BC treatments (3). In a systematic review and meta-analysis by Lavín-Pérez et al. (2023), researchers investigated the impact of PE on tumor-specific immune cells (CD4+ and CD8+) in BC patients (4). Their findings indicated that PE did not have a significant effect on these tumor-specific immune cells during cancer (4). However, the researchers emphasized that the limited number of studies in this field renders their conclusions preliminary and highlights the need for further research. However, exercise oncology studies have reported tumor shrinkage and cancer cell apoptosis induced by APA during BC (5, 6), but still limited immunological results are observed in this field. Several research gaps remain within the field of exercise oncology that need to be addressed. The investigation into the immunological changes of tumor-specific immune cells (CD4+ and CD8+) due to PE in BC patients is limited and yields contradictory results.

Although recent preclinical and clinical evidence — such as that of Gomes-Santos et al. (7) and Kurtz et al. (8) reported that aerobic training combined with PDL−1 blockade increased T cell infiltration the interaction between physical exercise and immune−checkpoint blockade therapies, particularly in melanoma and pancreatic cancer, the mechanistic understanding within breast cancer remains limited (9, 10). Specifically, the effects of controlled aerobic exercise combined with PD-L1 immune checkpoint blockadePD−L1 have not been systematically examined in murine models through the lens of exercise immunology. PD−L1. Recent investigations have provided mechanistic evidence that structured physical exercise can potentiate antitumor immune responses in solid tumors. For instance, Pedersen et al. (11) demonstrated exercise-induced mobilization of cytotoxic T cells and NK cells into tumor sites in melanoma models. Similarly, Kurtz et al. (8) reported enhanced checkpoint blockade efficacy following endurance exercise in pancreatic cancer. In breast cancer, Gomes-Santos et al. (7) highlighted how aerobic training synergized with PD−L1 blockade to improve T cell infiltration and tumor suppression. Therefore, the aim of the present study was to investigate whether structured aerobic exercise, when combined with PD−L1 immune checkpoint blockade, is associated with alterations in intratumoral CD4^+^ and CD8^+^ T−cell infiltration and tumor growth in a murine model of breast cancer. Specifically, we sought to compare the effects of exercise performed before tumor induction, during tumor progression, and their combination with anti−PD−L1 antibody administration, in order to better characterize how exercise timing may interact with immune checkpoint therapy in an immunocompetent breast cancer model.

## 2 Materials and methods

The Ethics Committee in Biomedical Research of Tarbiat Modares University of Tehran (IR.MODARES.AEC.1403.023) approved this study. All animal procedures were conducted in compliance with the guidelines for the care and use of laboratory animals and ethical principles in animal research as endorsed by the Iranian Council for the Control of Animal Experiments. This study adheres to the ARRIVE guidelines.

### 2.1 Animals

Thirty female mice, weighing between 18–20 grams and approximately at 4 weeks of age, were purchased from the mouse-breeding center of Rafsanjan University of Medical Sciences. They were housed in special Plexiglas cages under controlled environmental conditions, with an average temperature of 22 ± 1.4 °C, humidity of 50 ± 4%, and a 12-hour light-dark cycle. The mice had free access to the laboratory animal food and water. At the beginning of the experiment, mice underwent a 4−day adaptation period to become accustomed to the treadmill. Following adaptation, they were trained three times per week for 60 minutes per session during the subsequent exercise protocols. Five cancer-afflicted mice and five healthy mice warmed up for 10 minutes and began training at a speed of 6 m/min. The mice were habituated to the treadmill for four consecutive days before treadmill performance testing. The adaptation period involved 5 minutes of running at a speed of 6 m/min, followed by 5 minutes at speeds ranging from 6 to 12 m/min. For the exercise test, the mice warmed up for 3 minutes at a speed of 6 m/min. Then, they trained at a speed of 3 m/min for 3 minutes with no incline, and every 3 minutes, the treadmill speed was increased by 3 m/min until the mice reached fatigue (12–15). Finally, the exercise-training program was formulated based on the maximum running speed achieved by the mice. The chosen inoculation site (upper right thigh, corresponding to the posterior mammary fat pad) allows standard modeling of breast tumors while minimizing potential interference with treadmill movement or injury to forelimb mammary sites, ensuring concurrent feasibility of longitudinal exercise tracking and immunological sampling.

### 2.2 Cell line

The 4T1 murine breast cancer cell line, originally established from a spontaneously arising mammary adenocarcinoma in BALB/c mice, was used in this study. The 4T1 cell line is highly tumorigenic, poorly differentiated, and exhibits a triple−negative–like phenotype. Importantly, it retains the ability to form primary tumors and spontaneous metastases in immunocompetent hosts, making it a well−established preclinical model for studying tumor–immune interactions. 4T1 cells were maintained in RPMI−1640 medium supplemented with 10% fetal bovine serum and 1% penicillin–streptomycin under sterile conditions at 37 °C in a humidified atmosphere containing 5% CO_2_. The culture medium was replaced every 48 hours until approximately 70% confluence was reached. Cells were detached using trypsin, washed with RPMI−1640 and PBS, counted, and resuspended at concentrations ranging from 5 × 10^5^ to 1 × 10^6^ cells/mL. For tumor induction, 4T1 cells were injected subcutaneously into the upper right thigh (lateral femoral region), anatomically corresponding to the posterior mammary fat pad. This cell line and implantation model were selected because they reliably establish tumors in immunocompetent mice while preserving intact immune responses, thereby providing a biologically appropriate platform for evaluating exercise−induced modulation of CD4^+^ and CD8^+^ T−cell infiltration and responses to PD−L1 blockade. The chosen injection site also minimized mechanical interference with treadmill−based exercise protocols.

### 2.3 Protocol 1 (exercise before cancer induction)

All groups, except for the PCG, underwent training for 60 minutes per session, 5 sessions per week, during the first 6 weeks without the induction of cancer or administration of antibodies. The mice were trained on a 5-lane treadmill.

### 2.4 Protocol 2 (exercise after cancer induction) + anti-PD−L1 injection

**Figure 1:**
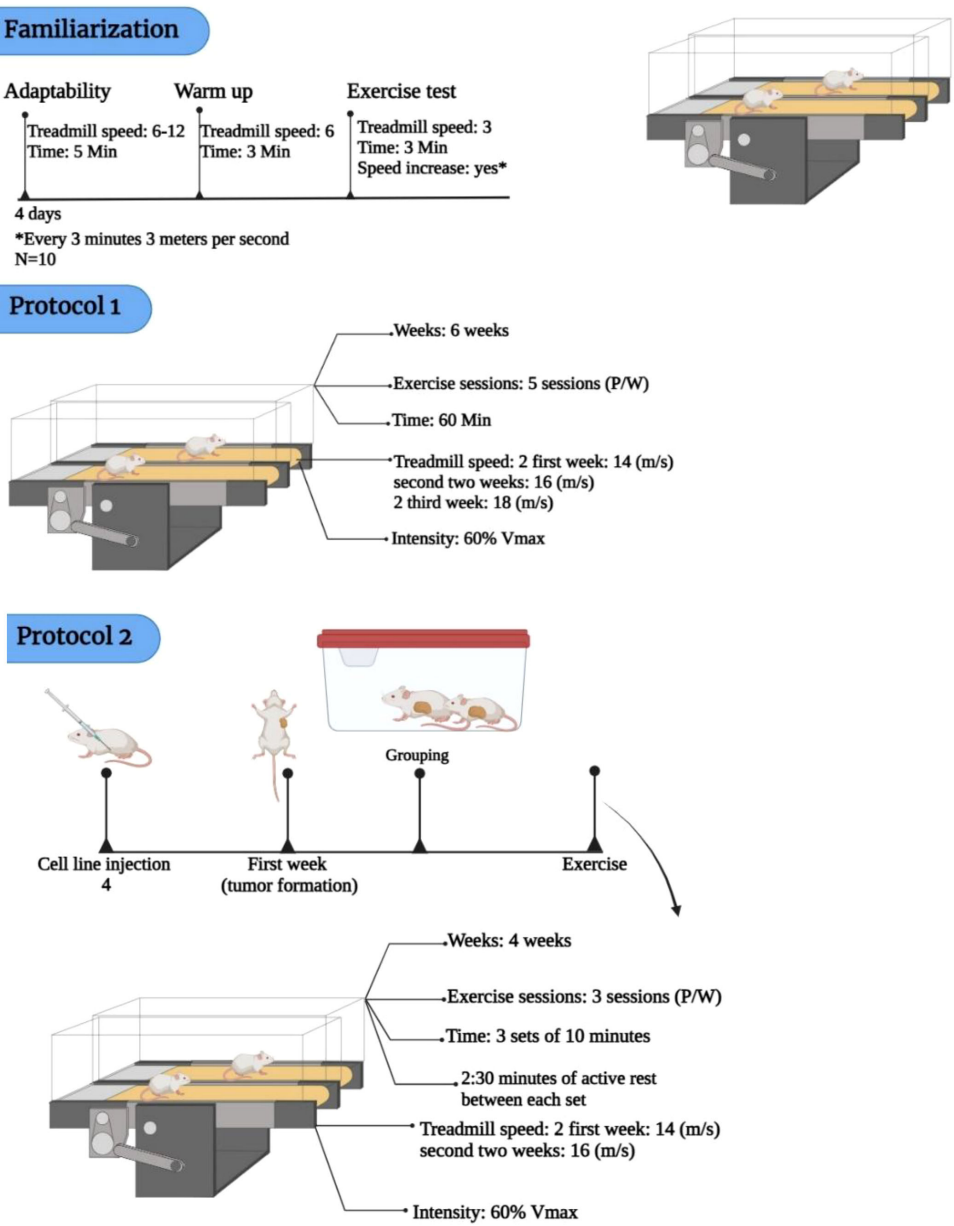
Schematic representation of the exercise training protocols.

After 6 weeks, breast tumors were induced by subcutaneous injection of 4T1 cells (1 × 10^6^ cells per mouse, suspended in 100 µL sterile PBS) into the upper right thigh region, anatomically corresponding to the posterior mammary fat pad. Two weeks after the cell line injection, the tumor was palpable at the injection site. Upon tumor appearance in the first week, the mice were randomly assigned into the following groups: PCG (N = 6), EIC (N = 6), EIE (N = 6), EIA (N = 6), and EIE+A (N = 6). The mice in the exercise groups trained three times per week, performing three 10−minute bouts at an intensity corresponding to ~60 % of each mouse’s individual maximal running velocity, with 2 minutes 30 seconds of active rest at ~40 % of maximal velocity between sets. The relative intensity (60 %) was recalculated every two weeks through a treadmill maximal running test to account for performance progression. The treadmill incline was kept at 0° throughout (14–16). The exercise intensity for each mouse was set at approximately 60 % of its individual maximal running velocity, determined by a treadmill exercise test performed at baseline. To ensure progressive and controlled overload, a new maximal running test was conducted every two weeks, and the training velocity was readjusted accordingly to maintain the relative 60 % workload. Therefore, the average velocity observed across sessions started at approximately 14 m/min and increased as the animals’ capacity improved, reflecting bi−weekly reassessments rather than a predefined linear increment (14–16). In the entire length of the research, the incline of the treadmill was zero. The PCG did not engage in any physical activity nor received antibodies after cancer induction (**Figure 1**). To examine the effect of exercise timing, two distinct but intensity-matched protocols were implemented. Protocol 1 (Pre-conditioning) consisted of aerobic treadmill training before tumor induction, and Protocol 2 (Therapeutic) was applied after tumor appearance. Both protocols were identical in structure and workload, each involving treadmill running at ~60 % of individual maximal velocity, with three 10-minute bouts separated by 2.5 minutes active recovery at ~40 %, performed three times per week for 60 minutes per session, with zero incline. A new maximal running test was conducted every two weeks to readjust individual velocities and maintain relative intensity. Consequently, the protocols differed solely by timing relative to tumor induction, allowing the assessment of how exercise performed before versus during cancer progression influences antitumor immunity.

In groups EIA and EIE+A, anti−PD−L1 antibody (clone 10F.9G2, Bio X Cell) was administered intraperitoneally at a dose of 10 μg per mouse, diluted in sterile PBS to a final volume of 100 μL per injection, once weekly for 4 doses. This dosage was selected based on pharmacokinetic and immunological evidence indicating that doses below 5 μg exhibit rapid clearance due to high-affinity binding of anti-PD−L1 antibodies to PD−L1 expressed on peripheral immune cells, a process described as Target-Mediated Drug Disposition (TMDD), in which drug clearance is governed by saturable binding to pharmacological targets (17, 18). At the 10 μg dose, saturation of these peripheral PD−L1 receptors occurs, resulting in linear pharmacokinetics and effective sustained tumor exposure (17–19). Gomes-Santos et al. (2021) (7) demonstrated that 10 μg weekly anti-PD−L1 combined with aerobic exercise maximizes CD8+ T-cell infiltration and antitumor synergy in 4T1 breast cancer models, whereas higher doses reduce the benefit of exercise-induced immune potentiation. Dose normalization calculations reveal that 10 μg per mouse (~400-500 μg/kg) corresponds to approximately 4–6 mg/kg human equivalent dose, consistent with clinical checkpoint inhibitor dosing ranges (18). These factors justify selecting 10 μg as an optimal, translationally relevant dose that synergizes with exercise-induced immune activation.

Protocol 1 – Pre−conditioning exercise before tumor induction (6 weeks), Protocol 2 – Exercise during tumor progression (4 weeks). Both protocols were matched for intensity (~60 % of individual maximal velocity), duration (60 min/session), and frequency (3 sessions/week). Each session consisted of three 10−minute running bouts separated by 2.5 minutes of active recovery at ~40 % maximal velocity. Periodic maximal tests ensured maintenance of the relative intensity. This design allowed the mechanistic comparison of timing (before vs during tumor development) under identical workload conditions.

### 2.5 Tumor tissue processing and immunofluorescence quantification of CD4^+^ and CD8^+^ T cells

### 2.6 Histological and immunofluorescence analysis (immunofluorescence staining and controls)

Tumor sections (5 µm) were deparaffinized and rehydrated as standard, followed by antigen retrieval in citrate buffer (pH 6.0) at 95 °C for 20 min. Non−specific binding was blocked using 5% normal goat serum for 30 min at room temperature. No peroxidase blocking (H_2_O_2_) was applied, as the procedure used pure immunofluorescence (IF) detection rather than chromogenic IHC. Sections were incubated overnight (4 °C) with primary antibodies against CD4^+^ (D7D2Z) and CD8α^+^ (D4W2Z), both Rabbit mAbs (Cell Signaling Technology, USA), diluted 1:200 in PBS. Secondary detection utilized FITC−conjugated Goat Anti−Rabbit IgG (H+L) (Elabscience, USA, Cat No. E−AB−1014). To verify staining specificity, isotype−matched negative controls (Rabbit IgG isotype control; Cell Signaling Technology, #3900) and non−immune IgG controls were processed in parallel and imaged under identical exposure settings. These controls confirmed negligible background fluorescence. Slides were counterstained with DAPI, mounted in anti−fade medium, and imaged at 200× magnification (Microbin 5 camera; KoreaTek microscope). Image analysis and thresholding were performed in ImageJ 1.53a using identical settings across groups.

### 2.7 Quantification of CD8^+^ and CD4^+^ Immunostaining

Immunofluorescence images were analyzed using ImageJ 1.53a software. After converting images to 8-bit grayscale, an intensity threshold was applied to isolate positive staining (green = CD8^+^; CD4^+^). For each mouse, five randomly selected non-overlapping fields (200×) from different tumor regions were analyzed. The fraction of CD8^+^-positive area was calculated as:

The fraction of CD8^+^ positive area was calculated as:


$$\left(\text{Positive CD}8^{+}\text{ pixels}/\text{Total pixels}\right)\times 100.$$

Data are presented as mean ± SEM of percentage of positive area, normalized to the PCG group (fold change = value/PCG mean). Quantitative comparisons between groups were assessed by one-way ANOVA with *P* < 0.05 considered significant.

### 2.7 Reagents and antibodies

Antibodies used in this study were as follows: InVivoMAb anti−mouse PD−L1 (B7−H1; clone 10F.9G2™, Bio X Cell, USA; catalog no. BE0101), rabbit anti−mouse CD4 monoclonal antibody (clone D7D2Z, Cell Signaling Technology, USA; catalog no. 25229; UniProt ID: P06332), rabbit anti−mouse CD8α monoclonal antibody (clone D4W2Z, Cell Signaling Technology, USA; catalog no. 98941; UniProt ID: P01731), and FITC−conjugated goat anti−rabbit IgG (H+L) secondary antibody (Elabscience, USA; catalog no. E−AB−1014), RPMI−1640 medium (Gibco, Thermo Fisher Scientific, USA; catalog no. 11875−093), fetal bovine serum (FBS; Gibco, USA; catalog no. 10082−147), penicillin–streptomycin (Gibco, USA; catalog no. 15140−122), trypsin−EDTA (Gibco, USA; catalog no. 25200−056) and phosphate−buffered saline (PBS; Gibco, USA; catalog no. 10010−023).

### 2.8 Measurement of tumor tissue

Tumor growth was monitored once per week for four weeks after induction (Weeks 7–10 of the experimental timeline) using a digital caliper. For each animal, two perpendicular dimensions were measured: the largest diameter (L) and the perpendicular width (W). Tumor volume (V) was calculated using the standard ellipsoid formula (V = [π/6] × W × L²). All caliper measurements were performed by the same blinded investigator to minimize operator bias.

**Figure 2:**
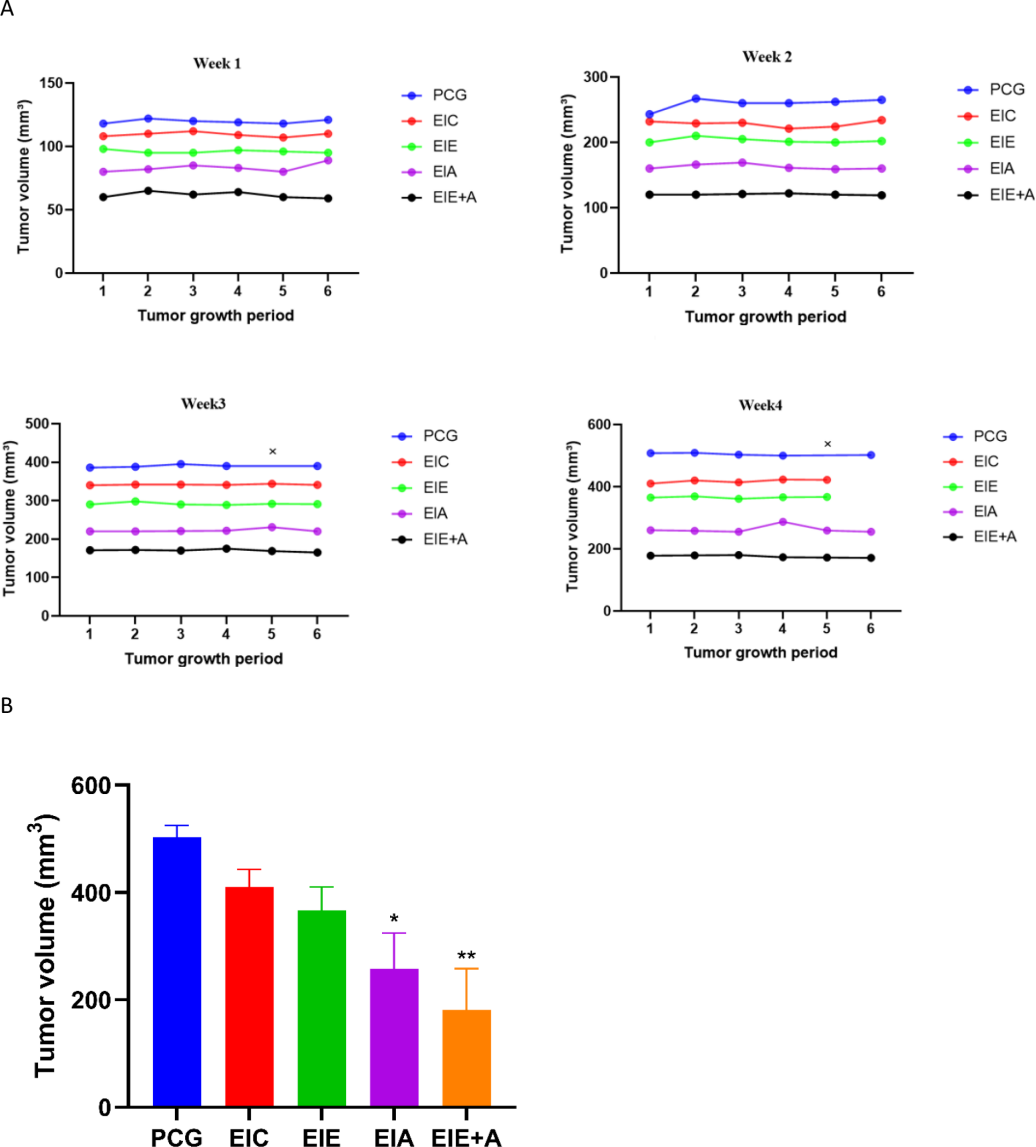
**(a)** Individual per-animal tumor growth trajectories during weeks 1–4 post-induction. Each panel shows tumor volume (mm³) for individual mice (n = 6/group) over sequential measurement points within the indicated week. Thin lines represent individual animals; bold lines represent group means. Animals euthanized early (censored) are marked with × at their final measurement point and trajectories truncated. Groups: PCG (blue), EIC (red), EIE (green), EIA (purple), EIE+A (black). Y-axis scales match each week’s range for clarity. Endpoint tumor volume means (Week 4) are presented in **(b)**. Final tumor burden and representative tumor morphology at standardized 10-week endpoint. Left panel: bar graph of final tumor weights (g) at Week 10 post-induction across all treatment groups (mean ± SEM, n=6). Right panel: representative tumor photographs showing morphology and relative size. All mice surviving to Week 10 were euthanized at this fixed timepoint to ensure equal treatment duration. For individual animal tumor volume trajectories, see **(a)**. ** The significance of the EIC with the PCG *** The significance of the EIE group with the PCG **** The significance of the EIA group with the PCG ***** The significance of the EIE+A group with the PCG.

Only the endpoint tumor volume (Week 10 post−induction) was used for statistical comparison among groups. The weekly trajectory shown in **Figure 2A** illustrates simulated mean growth patterns generated from these endpoint values to visualize inter−group variability.

Humane endpoint criteria.

All animals were observed daily for general health and tumor burden. Mice were euthanized immediately if any humane endpoint was reached, defined as:

– tumor size ≥ 2 cm in any dimension,
– > 15 % body−weight loss, or
– ulceration, necrosis, or impaired mobility.

To avoid time−dependent bias, all immunological analyses were performed at a fixed 10−week endpoint (post−induction). Mice that reached humane endpoints before Week 10 were humanely euthanized and included in the trajectory plot (**Figure 2A**) up to their last measurement but excluded from endpoint tissue quantification.

### 2.9 Attrition and data inclusion

Each experimental group contained 6 mice (n = 6). Minor attrition (1–2 mice in the PCG group) occurred due to heavy tumor load. No animals were excluded for non−biological reasons. Endpoint variability remained within ± 10 % across groups. Tumor tissues collected at the standardized Week 10 endpoint were processed for CD4^+^ and CD8^+^ immunofluorescence analyses.

### 2.10 Data analysis

Descriptive statistics were used to summarize the data and are presented as mean ± SEM. All statistical analyses were performed using IBM SPSS Statistics version 24.0, with statistical significance set at P < 0.05. Data normality was assessed using the Kolmogorov–Smirnov test. Group comparisons were conducted using one-way analysis of variance (ANOVA). When a significant overall effect was detected, *post hoc* multiple-comparison analysis was performed using Tukey’s test to identify differences between specific groups.

## 3 Results

In the present study, we investigated the pathological changes in tumor tissue from mice with breast cancer following the completion of a training protocol. To assess the effects of physical activity and the anti−PD−L1 antibody on tumor-specific immune cells (CD4+ and CD8+), we conducted histological examinations of the tumor tissue using light microscopy. For quantification, we randomly photographed at least five distinct regions of each stained tissue section with a Microbin 5 camera and an optical microscope (KoreaTek). In each field, we selected areas with positive staining (red for antibodies conjugated with CY3 and green for antibodies conjugated with FITC). Using Microbin software, we calculated and reported the percentage of positive areas across different fields as Mean ±.

### 3.1 Effects of exercise and anti−PD−L1 treatment on CD4^+^ T−cell infiltration

**Figure 3:**
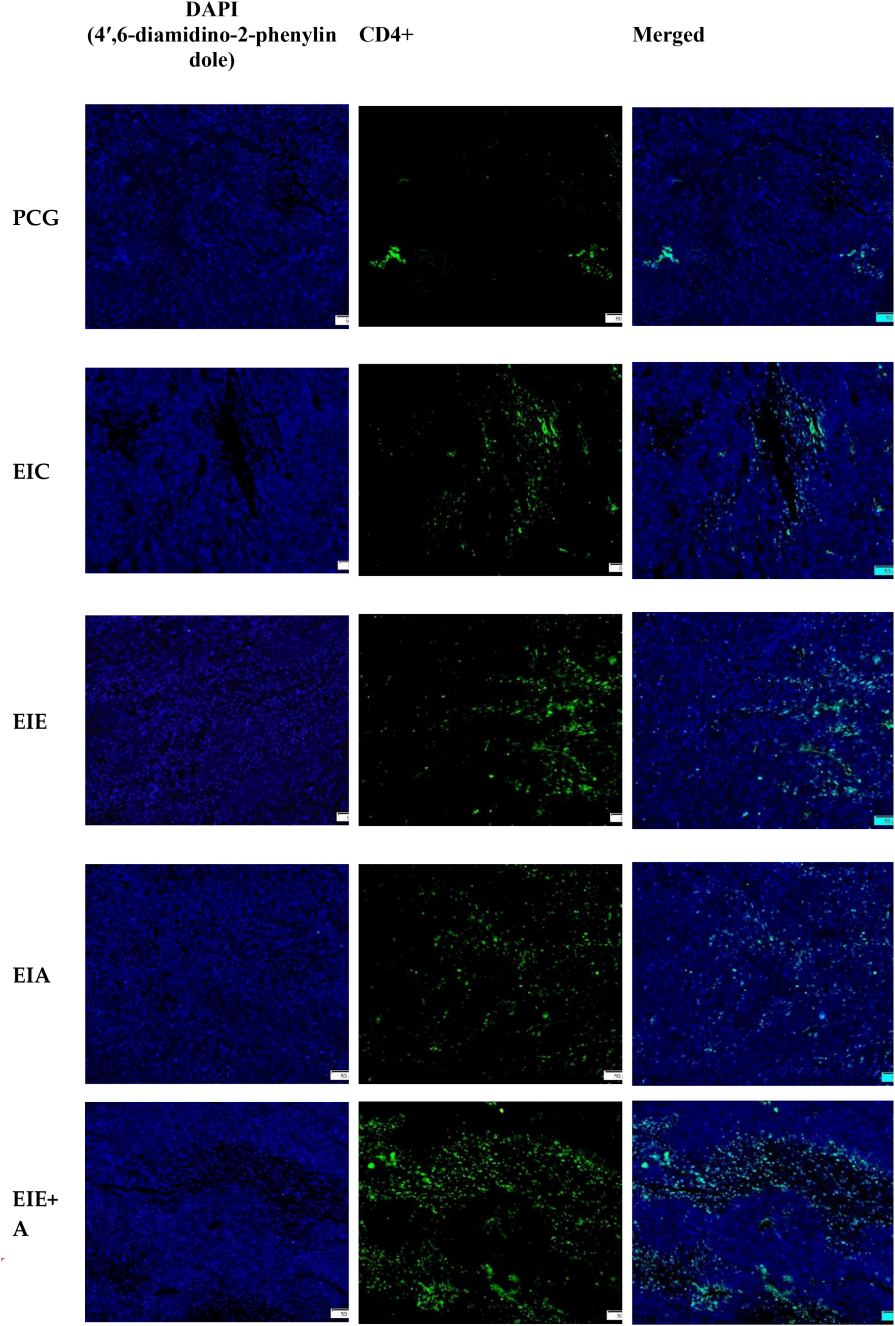
Representative immunofluorescence micrographs and quantitative analysis of CD4^+^ T−cell infiltration in tumor tissue.** Images captured at 200× magnification; scale bar = 50 µm. Data represent **mean ± SEM of % positive area per animal** (n = 6 mice/group). Fold change relative to PCG displayed on bars. *P < 0.05 vs PCG; one−way ANOVA.*.

**Figure 4:**
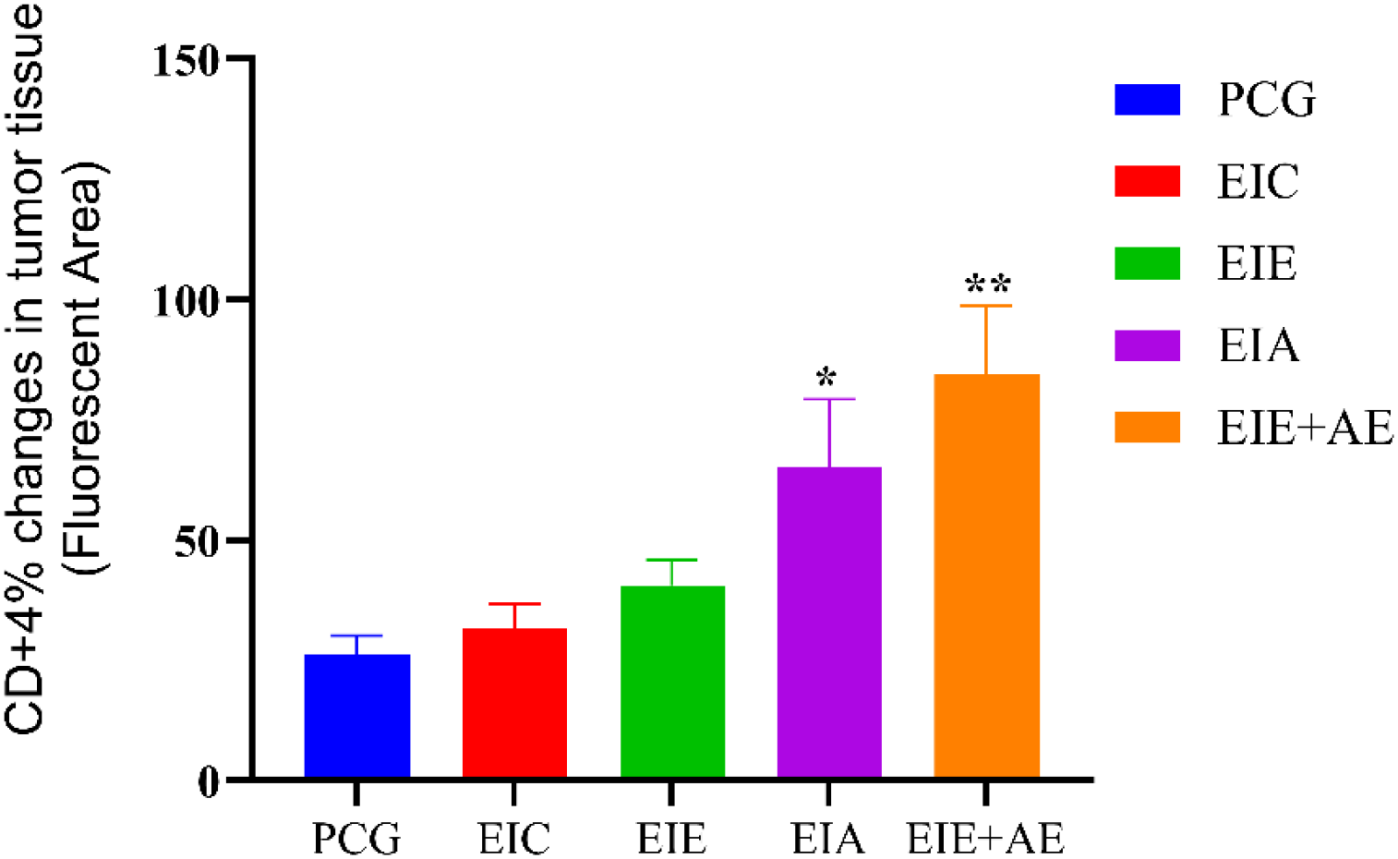
Quantitative analysis of CD4^+^ immune cell infiltration within tumor tissue. Data represent mean ± SEM of % CD4^+^−positive area (n = 6 mice/group), normalized to PCG (fold change shown on bars). One−way ANOVA; P < 0.05.*. * The significance of the EIA with the PCG ** The significance of the EIE+AE group with the PCG.

Representative immunofluorescence micrographs demonstrating CD4^+^ T−cell infiltration within tumor tissue are shown in **Figure 3**. Quantitative analysis revealed a gradual increase in CD4^+^ T−cell infiltration across the experimental groups. Mean ± SEM percentages of CD4^+^−positive area were 26.12 ± 2.11 % in PCG, 31.62 ± 2.34 % in EIC, 40.64 ± 3.05 % in EIE, 68.70 ± 4.22 % in EIA, and 83.12 ± 4.60 % in the EIE + A group. One−way ANOVA demonstrated a significant overall group effect on CD4^+^ T−cell infiltration. Subsequent Tukey’s *post hoc* analysis indicated that CD4^+^ infiltration was significantly higher in the EIA and EIE + A groups compared with the PCG group (P < 0.05). The combined exercise and anti−PD−L1 intervention (EIE + A) showed the most pronounced increase in CD4^+^ T−cell infiltration (**Figure 4**). These findings suggest that exercise training enhances CD4^+^ immune cell recruitment to tumor tissue, an effect that is further amplified when combined with PD−L1 blockade.

### 3.2 Effects of exercise and anti-PD-L1 treatment on CD8^+^ T-cell infiltration

**Figure 5:**
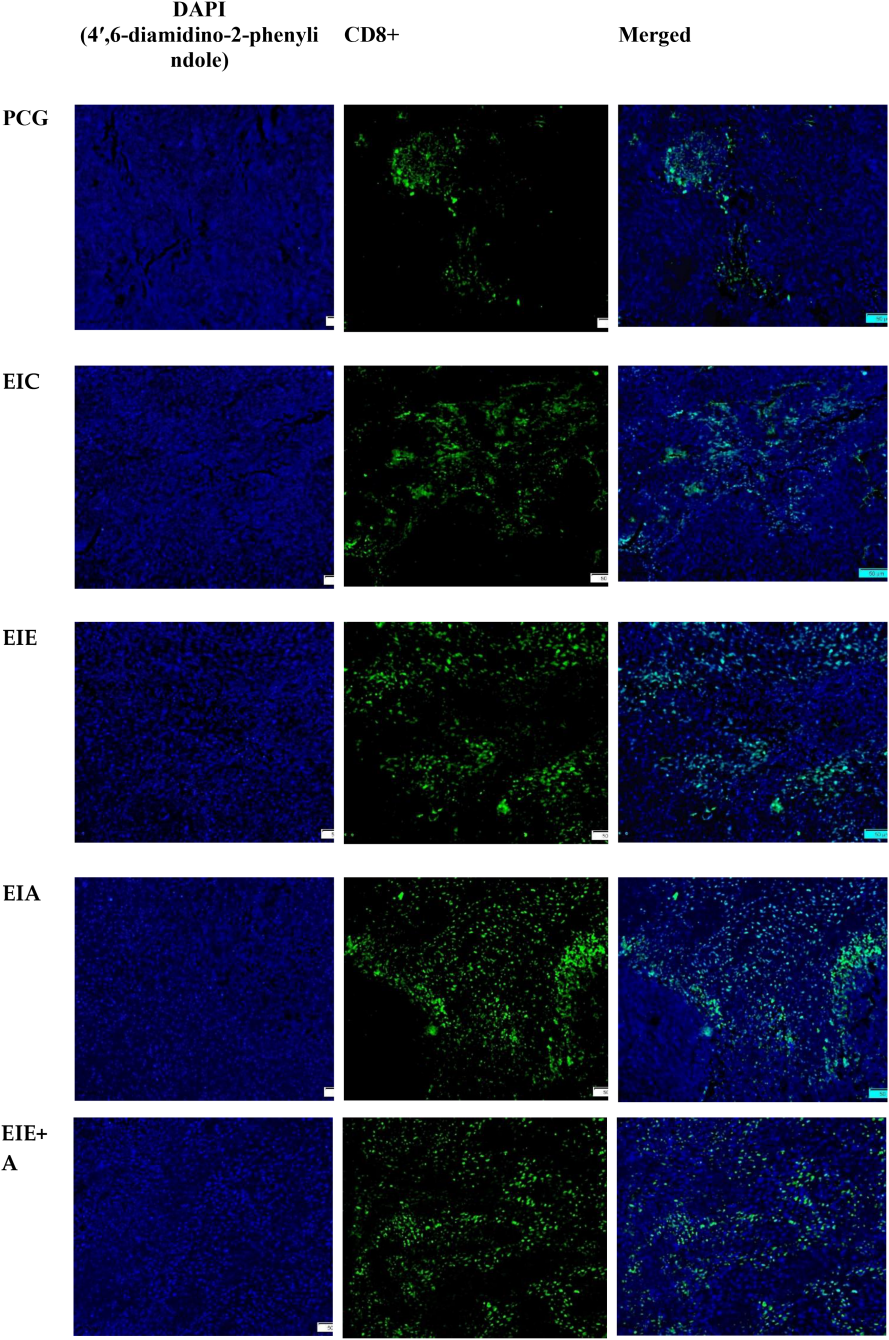
Representative immunofluorescence micrographs and quantitative analysis of CD8^+^ T−cell infiltration in tumor tissue of mice. Images captured at 200× magnification; scale bar = 50 µm. Data represent **mean ± SEM of % positive area per animal** (n = 6 mice/group). Fold change relative to PCG displayed on bars. *P < 0.05 vs PCG; one−way ANOVA.*.

**Figure 6:**
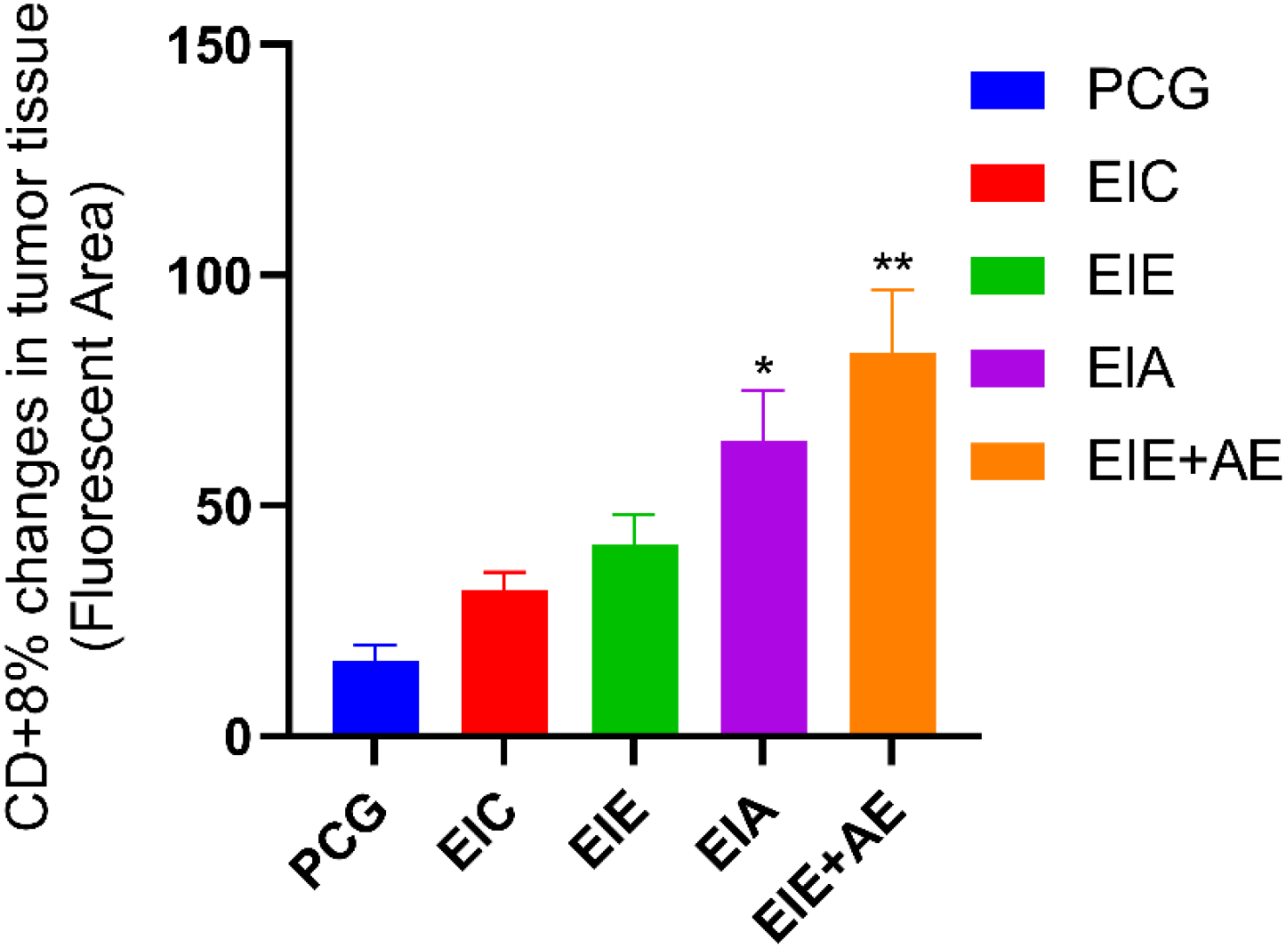
Quantitative analysis of CD8^+^ immune cell infiltration within tumor tissue. Values are mean ± SEM (% positive area; n = 6 mice/group). Fold change vs PCG shown on bars. One−way ANOVA; P < 0.05.* * The significance of the EIA with the PCG ** The significance of the EIE+AE group with the PCG.

CD8^+^ T−cell infiltration in tumor tissue was assessed by immunofluorescence analysis (**Figure 5**). Quantitative evaluation demonstrated a marked increase in CD8^+^ T−cell infiltration across intervention groups compared with the control group. The mean ± SEM percentages of CD8^+^−positive area were 16.52 ± 1.62 % for PCG, 26.56 ± 2.10 % for EIC, 45.31 ± 3.31 % for EIE, 64.12 ± 3.85 % for EIA, and 84.10 ± 4.53 % for the EIE + A group. One−way ANOVA revealed a significant effect of group on CD8^+^ T−cell infiltration. Tukey’s *post hoc* multiple−comparison test confirmed that CD8^+^ infiltration was significantly higher in the EIA and EIE + A groups compared with PCG (P < 0.05), with the greatest increase observed in the combined intervention group (**Figure 6**). These results indicate that the combination of exercise training and anti−PD−L1 therapy synergistically enhances cytotoxic T−cell infiltration into the tumor microenvironment.

### 3.3 Ratio of CD8^+^/CD4^+^ T-cell infiltration in tumor tissue

**Figure 7:**
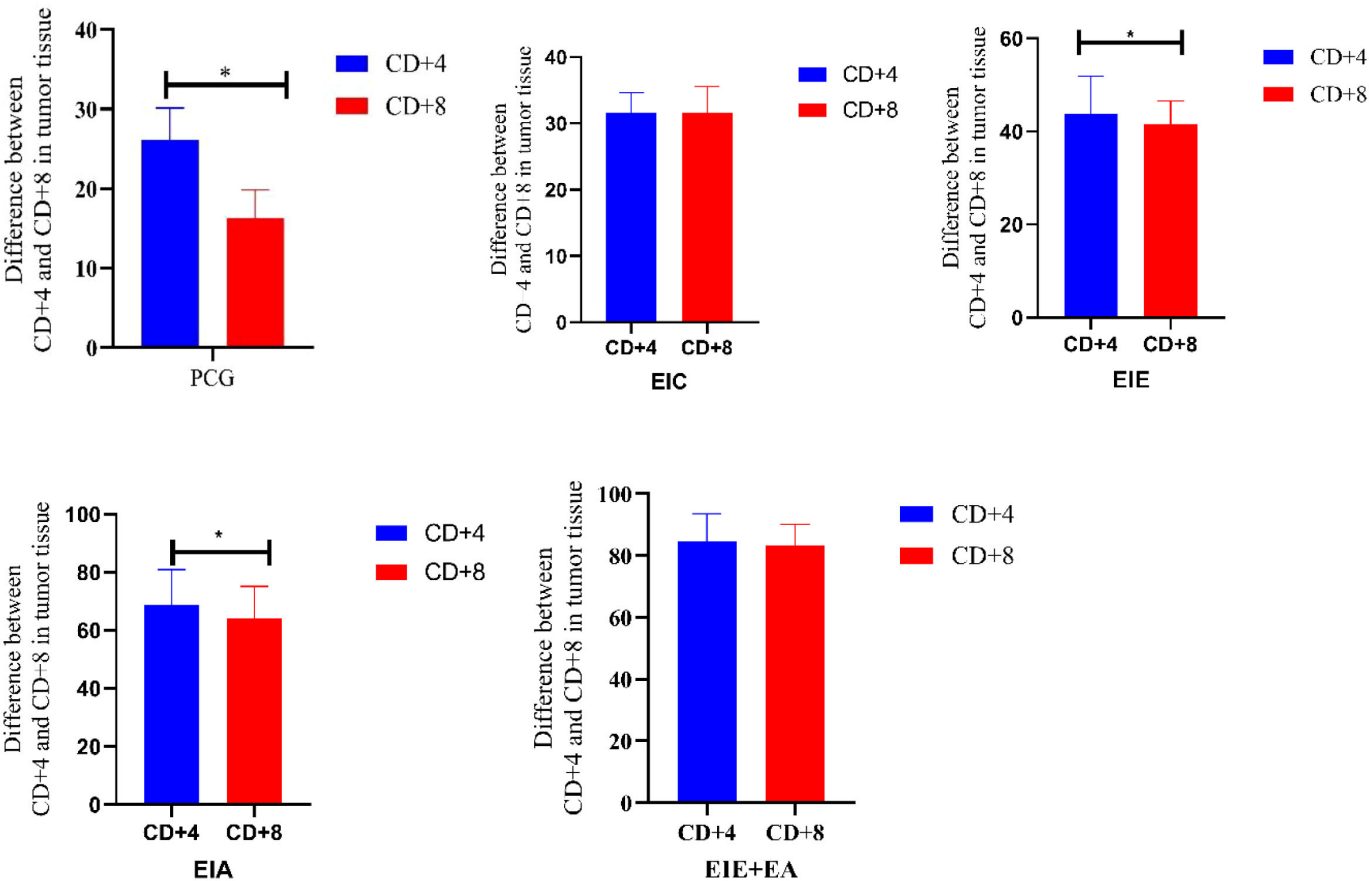
Ratio of CD8^+^/CD4^+^ T−cell infiltration within tumor tissue. Values represent mean ± SEM (n = 6 mice/group) based on percentage of positive area quantified by ImageJ 1.53a. Statistical analysis was performed using one−way ANOVA (P < 0.05). Higher ratio values indicate predominance of cytotoxic CD8^+^ T−cells relative to helper CD4^+^ T−cells.

To assess the balance between cytotoxic and helper T-cell infiltration within the tumor microenvironment, the ratio of CD8^+^ to CD4^+^ T cells was calculated based on quantitative immunofluorescence analysis (**Figure 7**). One-way ANOVA revealed a significant overall group effect on the CD8^+^/CD4^+^ ratio. Subsequent Tukey’s *post hoc* analysis demonstrated that groups receiving anti-PD-L1 in combination with exercise exhibited significantly higher CD8^+^/CD4^+^ ratios compared with control or exercise-only groups (P < 0.05). Specifically, the CD8^+^/CD4^+^ ratio was markedly increased in the EIA (2.46 ± 0.18) and EIE + A (2.80 ± 0.21) groups relative to PCG (0.63 ± 0.07), EIC (0.84 ± 0.09), and EIE (1.11 ± 0.12). The highest ratio was observed in the combined exercise and anti-PD-L1 group, indicating a shift toward a cytotoxic-dominant immune profile within tumor tissue. These findings suggest that the combined intervention preferentially promotes cytotoxic T-cell infiltration relative to helper T cells, thereby enhancing antitumor immune potential.

### 3.4 Comparison of CD4+ and CD8+ in tumor tissue all groups relative to the EIC group

**Figure 8:**
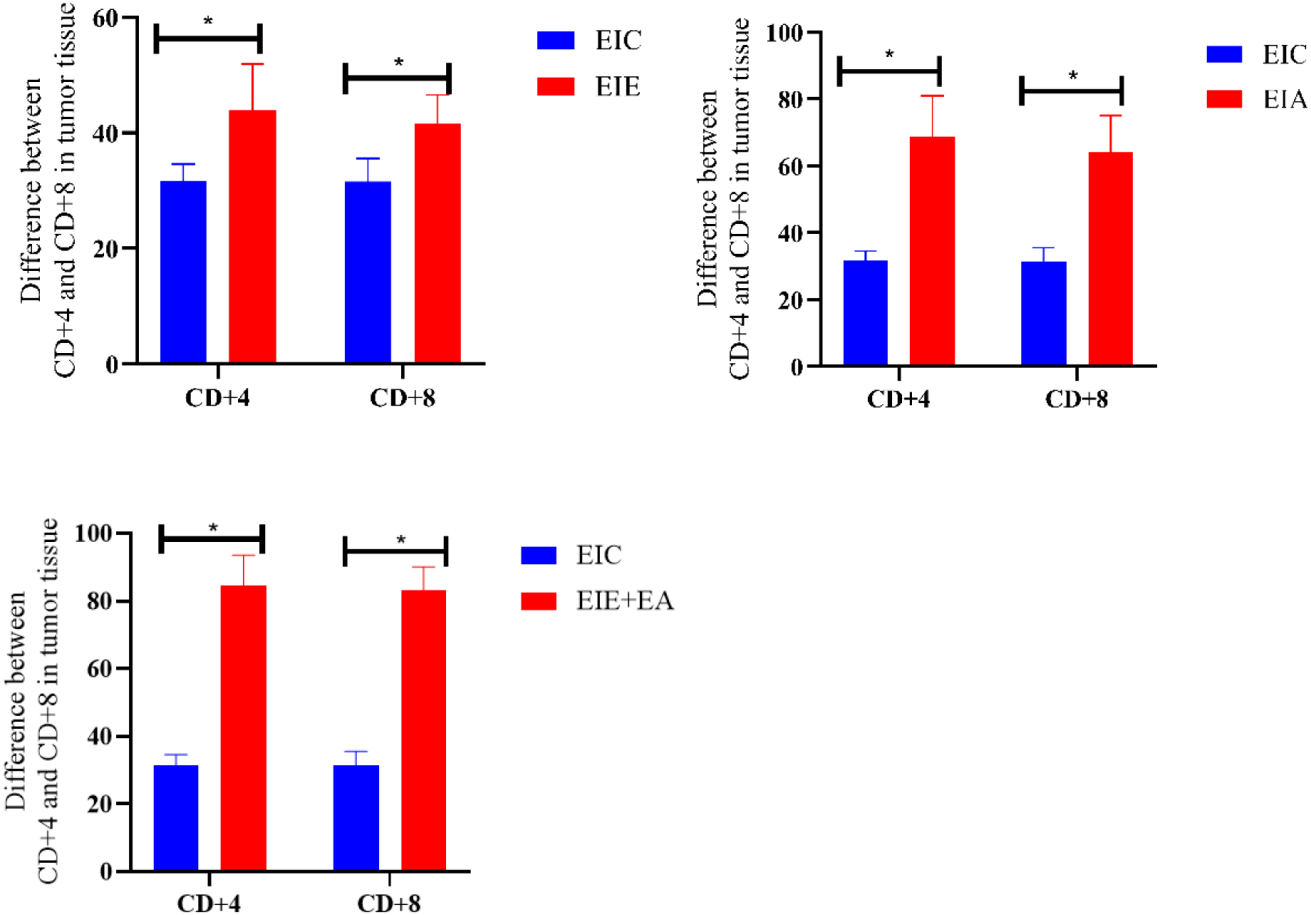
CD4+ and CD8+ in tumor tissue changes in between the EIE, EIA and EIE+A groups compared to the EIC.

To further evaluate treatment−associated differences in immune cell infiltration, CD4^+^ and CD8^+^ T−cell infiltration levels in the EIE, EIA, and EIE + A groups were compared with those in the EIC group (**Figure 8**). One−way ANOVA revealed a significant overall group effect. Subsequent Tukey’s *post hoc* analysis demonstrated that both CD4^+^ and CD8^+^ T−cell infiltration were significantly increased in the EIE group compared with the EIC group (P < 0.05). Similar significant increases were also observed in the EIA and EIE + A groups relative to EIC (P < 0.05) (**Figure 8**).

**Figure 9:**
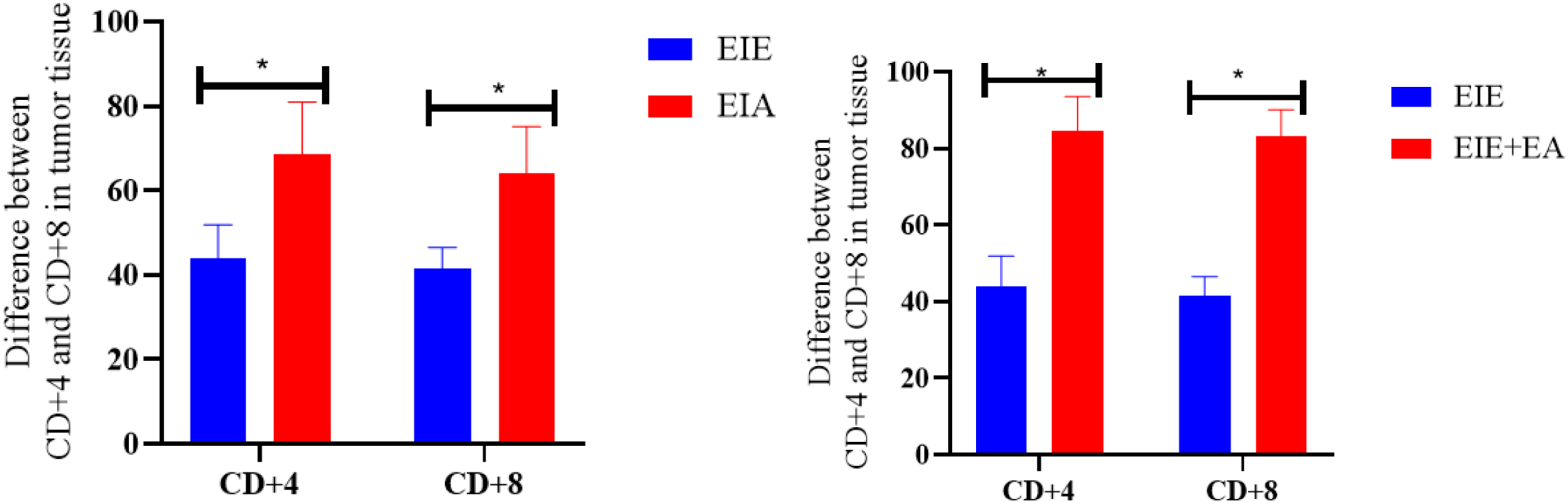
Quantitative analysis of CD8^+^ cell infiltration in tumor tissue. Values indicate mean ± SEM of % positive area (n = 6 mice/group) normalized to PCG. Fold change relative to PCG displayed on bars. P < 0.05 vs PCG by one−way ANOVA.

Quantitative comparison of immune infiltration across exercise and control groups is shown in **Figure 9**. The revised figure depicts quantitative CD8^+^ T-cell infiltration normalized to the non-exercise control group (PCG). Exercise alone (EIC and EIE) resulted in a modest increase in CD8^+^ infiltration compared with PCG, whereas combination interventions (EIA and EIE + A) produced significantly greater increases (P < 0.05). Data are presented as mean ± SEM percentage of positive area per field (n = 6 mice/group) and were analyzed using one-way ANOVA followed by Tukey’s *post hoc* test.

### 3.5 Comparison of CD4+ and CD8+ in tumor tissue all groups relative to the EIA group

**Figure 10:**
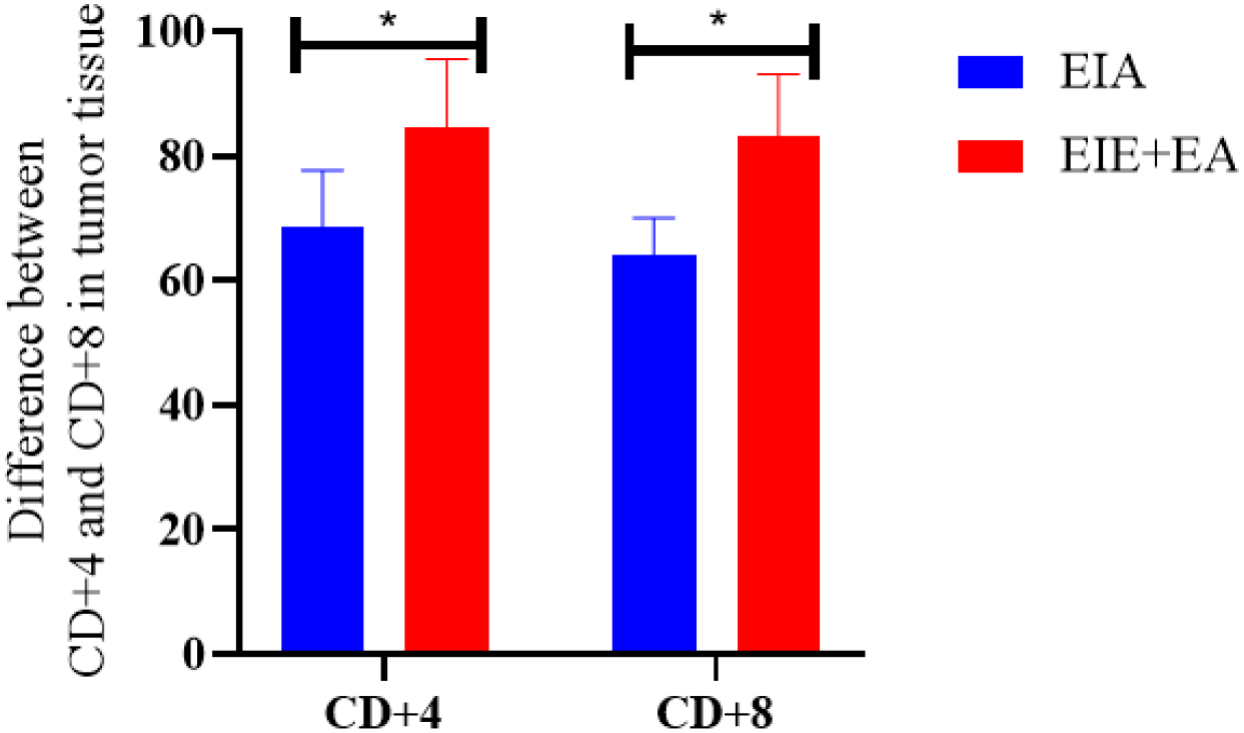
Quantitative analysis of CD4^+^ cell infiltration in tumor tissue. Data expressed as mean ± SEM % positive area (n = 6 mice/group) normalized to PCG. Fold change vs PCG shown on bars. P < 0.05 vs PCG by one−way ANOVA.

This section compares CD4^+^ and CD8^+^ T-cell infiltration between the EIA and EIE + A groups. One-way ANOVA revealed a significant overall group effect, and subsequent Tukey’s *post hoc* analysis demonstrated that CD4^+^ and CD8^+^ T-cell infiltration were significantly higher in the EIE + A group compared with the EIA group (P < 0.05) (**Figure 10**).

## 4 Tumor growth kinetics and final tumor weight

Tumor growth was monitored once weekly from Weeks 7 to 10 post-induction. As illustrated in **Figure 2A**, mean tumor-volume trajectories revealed a progressive increase in tumor size across all experimental groups. Endpoint analysis at Week 10 (**Figure 2B**) demonstrated significantly smaller tumor volumes in the EIA and EIE + A groups compared with the PCG. Mean ± SEM tumor volumes at Week 10 were 1642 ± 110 mm³ (PCG), 1328 ± 95 mm³ (EIC), 1085 ± 82 mm³ (EIE), 745 ± 66 mm³ (EIA), and 612 ± 55 mm³ (EIE + A). One-way ANOVA revealed a significant overall group effect, and Tukey’s *post hoc* analysis confirmed significant reductions in tumor volume in the EIA and EIE + A groups relative to PCG (P < 0.05). Final tumor weights measured at sacrifice followed a similar pattern, with significantly lower weights observed in the EIA (0.91 ± 0.07 g) and EIE + A (0.73 ± 0.06 g) groups compared with PCG (1.92 ± 0.11 g) (P < 0.05), as determined by one-way ANOVA followed by Tukey’s *post hoc* test. Together, the longitudinal growth trajectories and endpoint analyses indicate that the combination of physical activity and anti-PD-L1 antibody administration was associated with a marked attenuation of tumor growth compared with the non-exercise control group (see Supplementary Material 1).

## 5 Discussion

The present study was designed to evaluate the immunological and tumor−related effects of structured aerobic exercise, alone or in combination with PD−L1 immune checkpoint blockade, in a murine model of breast cancer. Specifically, we investigated how different timings of physical activity relative to tumor induction interact with anti−PD−L1 antibody administration to influence intratumoral CD4^+^ and CD8^+^ immune−cell infiltration and tumor growth. Results indicated that, in the EIC group compared to the PCG, physical activity appeared to influence the levels of CD4+ and CD8+ proteins in tumor tissue; however, this effect did not reach statistical significance. Notably, there was an observable increase in CD4+ and CD8+ expression in mice that engaged in physical activity during the cancer progression. In the EIE group versus the PCG, physical activity was associated with a rise in CD4+ and CD8+ protein levels in tumor tissue, although this increase was also not statistically significant. Nonetheless, the resumption of physical activity during the course of cancer treatment showed a marked effect on enhancing CD4+ and CD8+ expression compared to the EIC group. In the EIA group, when compared to the PCG, findings suggested that prior physical activity combined with anti−PD−L1 antibody treatment during cancer significantly contributed to an increase in CD4+ and CD8+ levels. The data imply that engaging in physical activity before the onset of cancer and administering anti−PD−L1 antibody during treatment was associated with higher expression of these immune markers compared to control in tumor tissue. Finally, in the EIE + Antibody group compared to the PCG, results demonstrated that both prior physical activity and the administration of anti−PD−L1 antibody during cancer treatment significantly elevated CD4+ and CD8+ levels. Overall, these findings suggest that physical activity, in conjunction with the administration of anti−PD−L1 antibody, was associated with increased CD4^+^ and CD8^+^ protein levels in tumor tissue in tumor tissue. Additionally, a significant reduction in tumor size was observed across all experimental groups relative to the PCG, indicating that both physical activity and anti−PD−L1 antibody administration may play a critical role in tumor size reduction. We have now included full growth−curve data and individual measurements for each animal, which confirm the reported statistical outcomes and provide clearer visualization of treatment−related trends.

In the present study, it was observed that physical activity, combined with the injection of an anti−PD−L1 antibody, can significantly increase CD4+ and CD8+ proteins within tumor tissue. It has been established that tumor growth is primarily regulated by CD4+ and CD8+ cells. Recently, there has been growing recognition that CD4+ T cells and CD8+ cells play a vital role in establishing and maintaining effective antitumor immunity. These cells mediate antitumor immunity not only through antibody receptors but also by secreting effector cytokines such as interferon-γ (IFN-γ) and tumor necrosis factor-α (TNF-α) under certain conditions (20, 21). However, in advanced stages of cancer, the performance of CD4+ and CD8+ cells is significantly impaired, which is one of the primary contributors to tumor growth and metastasis. Numerous studies have found that the decline in immune cell function is due to the binding of PD-1 to PD-L1. PD-1 is a cell surface receptor functioning as a T cell checkpoint and playing a key role in regulating T cell exhaustion. When PD-1 binds to its ligand,PD-L1, downstream signaling pathways are activated, leading to the inhibition of T cell activation (22, 23). This inhibition allows PD-L1 expressed on tumor cells to interact with PD-1 on T cells, leading to immune evasion by the tumor. Consistent with findings from the current study, several other studies have reported that administering anti-PD−L1 antibodies in cancer models can enhance the quantity and infiltration of CD4+ and CD8+ cells within tumor tissue. These studies revealed that doses ranging from 10 to 500 mg over varying intervals could effectively block PD-L1 expressed on tumors, prevent immune system evasion, and subsequently support CD4+ and CD8+ cell function in combating the tumor (24–28). In contrast, while physical activity was noted to influence CD4+ and CD8+ cell performance in tumor tissue, it did not produce a significant effect without anti−PD−L1 antibody injection. The present study demonstrated that physical activity alone could increase the permeability of CD4+ and CD8+ cells in tumor tissue; however, this effect became notably significant only when combined with anti−PD−L1 antibody administration. Limited research has examined this combined effect. A systematic review by Pérez and colleagues investigated the role of physical activity on tumor-specific immune cells (CD4+ and CD8+) during cancer, concluding that while evidence in this field is limited, physical activity appears not to have a negative impact on CD4+ and CD8+ function in cancer. Some studies, though limited, did indicate a positive effect (29). In line with these findings, the present study also showed an increase in CD4+ and CD8+ cell numbers in cancer models following physical activity.

In this study, it was found that physical activity combined with the injection of an anti−PD−L1 antibody can significantly reduce tumor volume or size. Tumor size reduction was observed to be significant in all experimental groups compared to the control group. Consistent with previous research, it has been shown that in cases where the immune system’s function is diminished, cancer tumors tend to grow rapidly to facilitate metastasis and expansion into surrounding tissue, leading to an increase in tumor size (4, 30–32). These studies support the findings of the present study in the PCG group. Additionally, studies utilizing either anti−PD−L1 antibody injections or physical activity alone have also demonstrated reductions in tumor size, aligning with the current findings (33–35). Results from these studies suggest that both physical activity and anti−PD−L1 antibody injections can enhance immune system function, particularly in CD8+ and CD4+ cells within tumor tissue (33–35). This increase in CD8+ and CD4+ cell activity is associated with the apoptosis of cancer cells and a corresponding reduction in tumor size.

**Figure 11:**
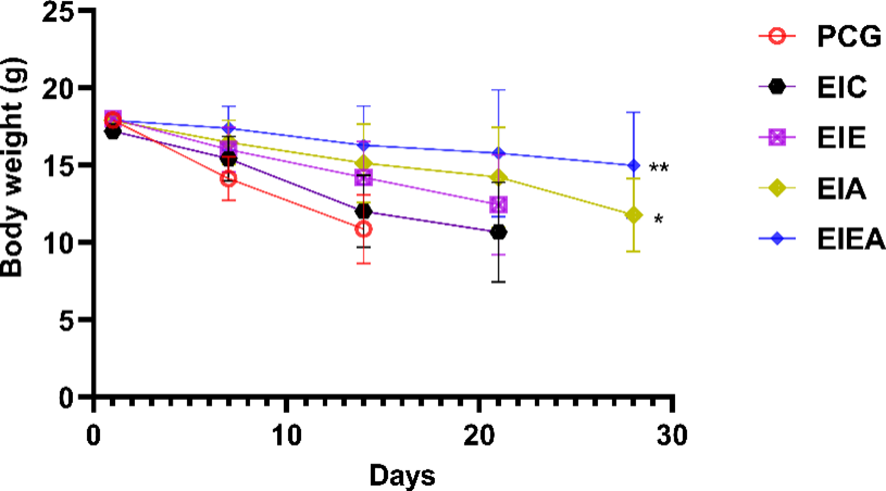
Body−weight change and survival profile across groups. Despite early mortality observed in the control group (PCG), all tumor tissues for quantitative immunological analysis were collected at the predefined 10−week endpoint to prevent time/size−dependent bias. Differences in survival therefore do not affect immune data interpretation.

**Figure 12:**
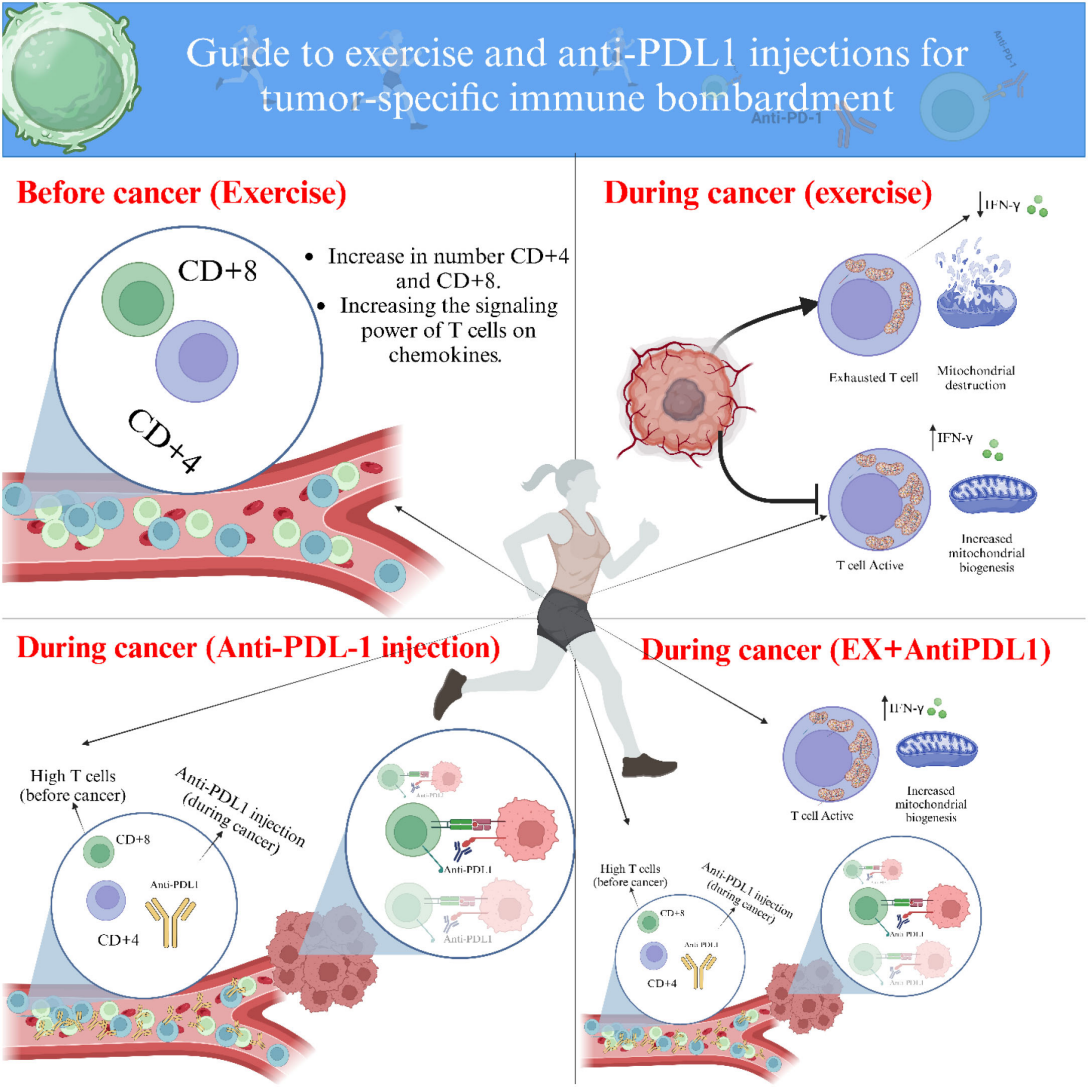
Schematic diagram of physical activity and anti-PDL1 injection.

A notable limitation of the present study is the absence of an examination of PD−L1 expression across different groups, as well as other immunological markers within tumor tissue, including both pro-inflammatory and anti-inflammatory cytokines. A major limitation that constrains the interpretation of the present findings is the absence of a critical control group consisting of mice treated with the anti−PD−L1 antibody alone, without any exercise intervention. Consequently, it is not possible to fully distinguish the independent immunotherapeutic contribution from the potential influence exerted by aerobic exercise. The significant effects observed in the EIA and EIE+A groups could therefore reflect, at least in part, the action of PD−L1 blockade itself. This limitation reduces the strength of causal inference regarding the combined, rather than isolated, effects of the two interventions. Future studies should incorporate a dedicated anti−PD−L1−only arm to clearly differentiate additive and interactions between physical exercise and immune checkpoint inhibition. Explicit inclusion of such a control will enable robust validation of the combinatorial hypothesis proposed in the current work.PD−L1. While the study included groups receiving combined interventions (i.e., exercise plus anti−PD−L1 antibody), the lack of an antibody-only group limits the ability to isolate the independent effects of PD−L1 blockade on tumor progression and immune responses. Including such a group would allow for a more precise comparison between the effects of exercise, immunotherapy, and their combination. Future studies should consider adding an anti-PD−L1-only group to better delineate the or additive effects of physical activity when used alongside immune checkpoint inhibitors. Another limitation of this study is the lack of cytokine profiling to support the immunological findings. Although increased CD4^+^ and CD8^+^ T cell infiltration was observed, the functional state of these cells remains unclear in the absence of cytokine data such as interferon-gamma (IFN-γ), tumor necrosis factor-alpha (TNF-α), or interleukins (e.g., IL-2, IL-10). These cytokines are critical indicators of T cell activation, immune polarization, and the tumor microenvironment’s inflammatory status. Future studies incorporating cytokine analysis at systemic and tumor levels would provide deeper insights into the mechanistic pathways underlying the immune-modulatory effects of physical activity and PD−L1 blockade. A further limitation is the use of a fixed antibody dose (10 µg/mouse) without normalization to body weight (**Figure 11**). While this approach is common in murine studies, especially when low-dose checkpoint blockade is explored, reporting dosage in mg/kg units could improve reproducibility and allow better comparison across studies. However, a key strength of this study lies in its focus on the immunological changes induced by the combined effects of anti−PD−L1 antibody injection and physical activity, with particular attention to their potential impact (**Figure 12**). A further mechanistic limitation concerns the functional characterization of the infiltrating T cells. Although this study demonstrated significant increases in CD4^+^ and CD8^+^ T-cell presence within tumor tissue, the functional activation status of these cells remains undetermined. Without assessment of effector cytokines such as interferon-γ (IFN-γ), tumor necrosis factor-α (TNF-α), and interleukins (e.g., IL-2), or cytolytic markers including granzyme B and perforin, it is not possible to conclusively discern whether the increased infiltration reflects active antitumor immunity or merely passive cell migration. Future studies should integrate cytokine profiling and intracellular functional assays to elucidate whether aerobic exercise in combination with PD−L1 blockade enhances the effector function and cytotoxic capacity of these tumor-infiltrating lymphocytes. Such analyses would provide a mechanistic bridge between immunological activation and the observed reduction in tumor burden. Another methodological limitation of the present study relates to the use of CD4 as a single immunohistochemical marker. It should be noted that CD4 is not exclusively expressed by T helper lymphocytes and may also label CD4^+^ macrophages and other antigen-presenting immune cells within the tumor microenvironment. Therefore, the CD4 immunostaining observed in this study reflects the infiltration of CD4^+^ immune cells rather than being strictly limited to CD4^+^ T cells. Additional phenotypic characterization using co-staining or flow cytometric approaches would be required to definitively distinguish CD4^+^ T lymphocytes from other CD4-expressing immune cell populations.

## Data Availability

The original contributions presented in the study are included in the article/supplementary material. Further inquiries can be directed to the corresponding authors.
