# Supplementary material for: Immuno−oncological effects of aerobic exercise combined with anti−PD−L1 antibody blockade in a murine breast cancer model
Source: Front Oncol. 2026 Jan 27;16:1671244. doi: 10.3389/fonc.2026.1671244 (PMC12888218; doi:10.3389/fonc.2026.1671244)
Supplement: Supplementary file 1 [file DataSheet1.docx]

Supplementary Material


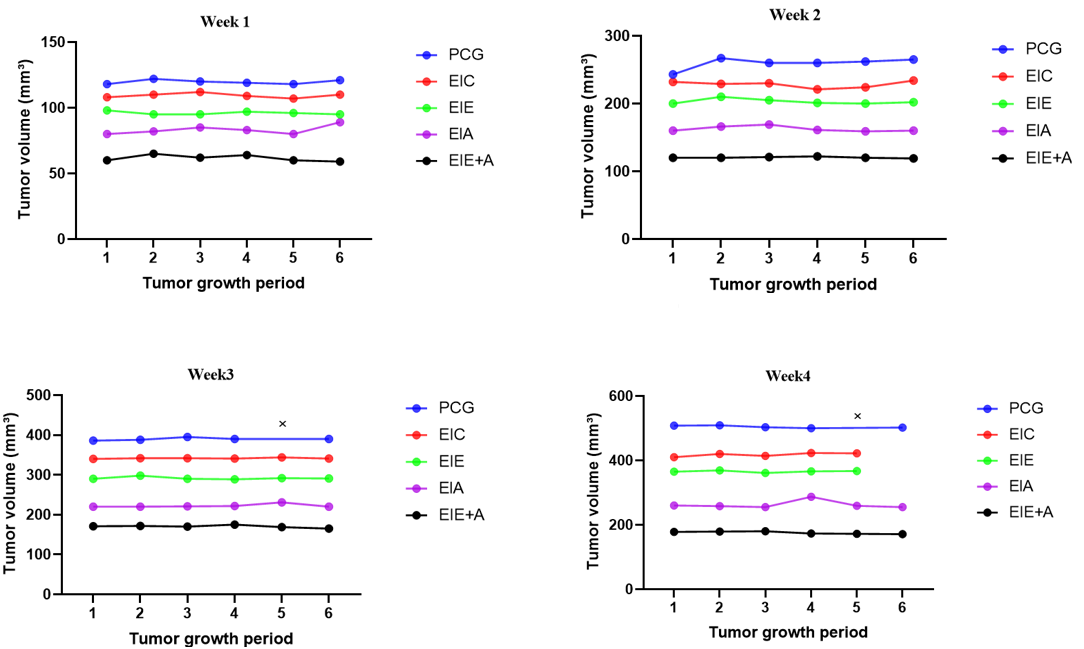


Figure 10a. Individual per-animal tumor growth trajectories during weeks 1–4 post-induction. Each panel shows tumor volume (mm³) for individual mice (n = 6/group) over sequential measurement points within the indicated week. Thin lines represent individual animals; bold lines represent group means. Animals euthanized early (censored) are marked with × at their final measurement point and trajectories truncated. Groups: PCG (blue), EIC (red), EIE (green), EIA (purple), EIE+A (black). Y-axis scales match each week’s range for clarity. Endpoint tumor volume means (Week 4) are presented in Figure 10b.


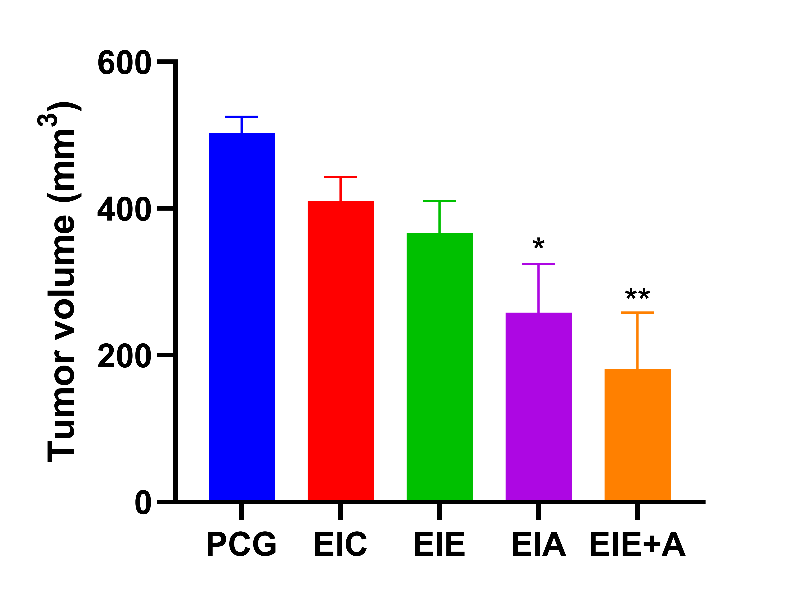


| PCG | EIC | EIE | EIA | EIE+A |
| --- | --- | --- | --- | --- |
| 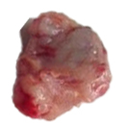 | 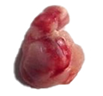 | 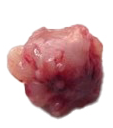 | 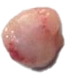 | 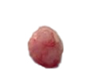 |
|  |  |  |  |  |
|  |  |  |  |  |

Figure 10b. Final tumor burden and representative tumor morphology at standardized 10-week endpoint. Left panel: bar graph of final tumor weights (g) at Week 10 post-induction across all treatment groups (mean ± SEM, n=6). Right panel: representative tumor photographs showing morphology and relative size. All mice surviving to Week 10 were euthanized at this fixed timepoint to ensure equal treatment duration. For individual animal tumor volume trajectories, see Figure 10a.

** The significance of the EIC with the PCG

*** The significance of the EIE group with the PCG

**** The significance of the EIA group with the PCG

***** The significance of the EIE+A group with the PCG


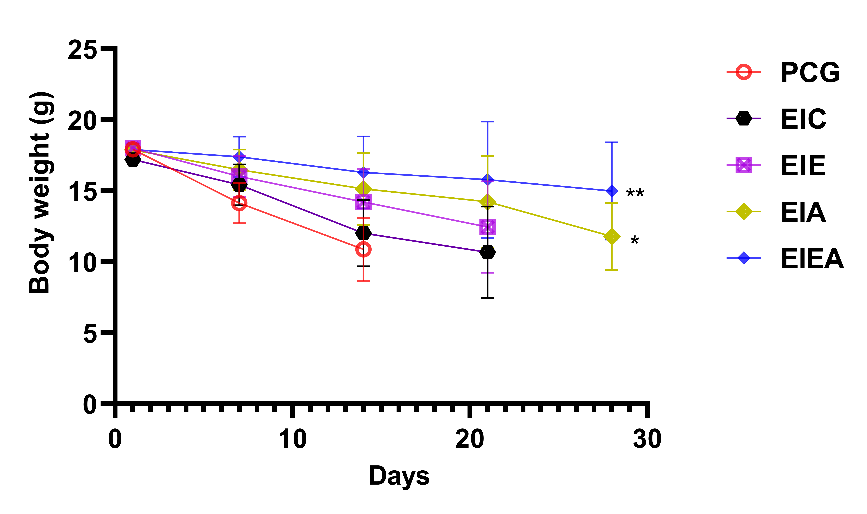


Figure11- Body‑weight change and survival profile across groups. Note: Despite early mortality observed in the control group (PCG), all tumor tissues for quantitative immunological analysis were collected at the predefined 10‑week endpoint to prevent time/size‑dependent bias. Differences in survival therefore do not affect immune data interpretation..
